# Patterns of recurrence after surgery and efficacy of salvage therapy after recurrence in patients with thoracic esophageal squamous cell carcinoma

**DOI:** 10.1186/s12885-020-6622-0

**Published:** 2020-02-22

**Authors:** Wenjie Ni, Jinsong Yang, Wei Deng, Zefen Xiao, Zongmei Zhou, Hongxing Zhang, Dongfu Chen, Qinfu Feng, Jun Liang, Jima Lv, Xiaozhen Wang, Xin Wang, Tao Zhang, Nan Bi, Lei Deng, Wenqing Wang

**Affiliations:** 1 Department of Radiation Oncology, National Cancer Center/National Clinical Research Center for Cancer/Cancer Hospital, Chinese Academy of Medical Sciences and Peking Union Medical College, No. 17 South Panjiayuan lane, Chaoyang District, Beijing, 100021 China; 2 Cancer Center, Union Hospital, Tongji Medical College, Huazhong University of Science and Technology, Wuhan, China; 3 Key Laboratory of Carcinogenesis and Translational Research (Ministry of Education/Beijing), Department of Radiation Oncology, Peking University Cancer Hospital & Institute, Beijing, China

**Keywords:** Esophageal neoplasm, Recurrence, Salvage treatment, Survival

## Abstract

**Background:**

Information on the optimal salvage regimen for recurrent esophageal cancer is scarce. We aimed to assess the patterns of locoregional failure, and evaluate the therapeutic efficacy of salvage therapy along with the prognostic factors in recurrent thoracic esophageal squamous cell carcinoma (TESCC) after radical esophagectomy.

**Methods:**

A total of 193 TESCC patients who were diagnosed with recurrence after radical surgery and received salvage treatment at our hospital were retrospectively reviewed from 2004 to 2014. The patterns of the first failure were assessed. The post-recurrence survival rate was determined using the Kaplan-Meier method and analyzed using the log-rank test. Multivariate prognostic analysis was performed using the Cox proportional hazard model.

**Results:**

The median time of failure was 7.0 months. Among the 193 patients, 163 exhibited isolated locoregional lymph node (LN) recurrence and 30 experienced locoregional LN relapse with hematogenous metastasis. Among the 193 patients, LN recurrence was noted at 302 sites; the most common sites included the supraclavicular (25.8%; 78/302) and mediastinal LNs (44.4%; 134/302), particularly stations 1 to 6 for the mediastinal LNs (36.4%; 110/302). The median overall survival (OS) was 13.1 months after recurrence. In those treated with salvage chemoradiotherapy, with radiotherapy, and without radiotherapy, the 1-year OS rates were 68.5, 55.0, and 28.6%; the 3-year OS rates were 35.4, 23.8, and 2.9%; and the 5-year OS rates were 31.8, 17.2, 2.9%, respectively (*P* < 0.001). Furthermore, patient survival in those who received salvage chemoradiotherapy was significantly better than those treated with salvage radiotherapy alone (*P* = 0.044). Multivariate analysis showed that the pathological TNM stage and salvage treatment regimen were independent prognostic factors.

**Conclusions:**

Supraclavicular and mediastinal LN failure were the most common types of recurrence after R0 surgery in TESCC patients. Salvage chemoradiotherapy or radiotherapy could significantly improve survival in esophageal cancer with locoregional LN recurrence.

## Background

Surgical resection is the mainstay for potentially curable esophageal cancer. However, locoregional recurrence (LRR) is the most common pattern of recurrence, and is noted in up to 23.8–58.0% of cases, whereas hematogeous metastasis is noted in approximately 5.5–33.0% of cases [1–6]. The median time to recurrence ranges from 7 to 12.5 months [7–9]. According to the National Comprehensive Cancer Network (NCCN) guidelines, salvage chemoradiotherapy is preferably recommended for such patients with recurrence. However, this recommendation is based on only a few retrospective studies with small sample sizes. Hence, the optimal recommendation for these patients remains unclear, and further studies on salvage treatment are required. In the present study, we aimed to retrospectively analyze the patterns of relapse and therapeutic efficacy of salvage therapy in our institution.

## Methods

### Eligibility criteria

The study enrolled patients who were diagnosed with LRR and who received salvage treatment at our institution after radical esophagectomy (R0) of TESCC. Patients who underwent neoadjuvant or adjuvant therapy were excluded.

### Patients and treatments

There were 193 patients eligible from January 2004 to December 2014, including 165 male and 28 female patients (median age, 60 years; mean age, 59.7 ± 8.6 years). Of these 193 patients, 163 exhibited locoregional LN recurrence alone and 30 exhibited locoregional LN relapse in combination with hematogenous metastases. After recurrence, 48 patients received salvage chemoradiotherapy (SCRT), 109 received salvage radiotherapy (SRT), and 36 received salvage chemotherapy or best supportive care (No RT) due to the presence of LN recurrence in multiple regions and/or hematogenous metastases (Table 1). Radiation therapy was delivered via a 6 MV X-ray linear accelerator. The median dose was 60 Gy (30 Gy in 1, 40–49 Gy in 6, 50–59 Gy in 45, 60–70 Gy in 103, and unknown in 2 patients). A total of 95 patients received involved field irradiation, whereas 60 patients underwent elective field radiotherapy. The most common chemotherapy regimen included platinum combined with taxane. Of the 66 patients received chemoradiotherapy or chemotherapy, there were 22 cases received paclitaxel plus nedaplatin, 25 received paclitaxel plus cisplatin, 9 received cisplatin plus 5-fluorouracil, and 10 received other regimens. Best supportive care involved nutritional support therapy, analgesic therapy, and anti-infective therapy. The main objective was to improve the quality of life.

**Table 1:** Patient demographic and clinical characteristics

	Number 193(%)	SCRT 48(%)	SRT 119(%)	No RT 36(%)	p
Sex					0.139
Male	165 (85.5)	45 (93.8)	89 (81.7)	31 (86.1)	
Female	28 (14.5)	3 (6.3)	20 (18.3)	5 (13.9)	
Age, years					0.054
≤ 60	100 (51.8)	32 (66.7)	50 (45.9)	18 (50.0)	
>60	93 (48.2)	16 (33.3)	59 (54.1)	18 (50.0)	
Location of the primary tumor					0.645
Upper	27 (14.0)	8 (16.7)	14 (12.8)	5 (13.9)	
Middle	91 (47.2)	25 (52.1)	52 (47.7)	14 (38.9)	
Lower	75 (38.9)	15 (31.3)	43 (39.4)	17 (47.2)	
Surgery method					<0.001
Sweet approach	148 (76.7)	10 (37.0)	67 (73.6)	71 (94.7)	
Ivor-Lewis approach	45 (23.3)	17 (63..0)	24 (26.4)	4 (5.3)	
Differentiation degree					0.488
Well	25 (13.0)	6 (12.5)	15 (13.8)	4 (11.1)	
Median	113 (58.5)	26 (54.2)	66 (60.6)	21 (58.3)	
Poor	54 (28.0)	16 (33.3)	28 (25.7)	10 (27.8)	
Unknown	1 (0.5)	–	–	–	
Pathological T stage*					0.097
T1	5 (2.6)	1 (2.1)	2 (1.8)	2 (5.6)	
T2	35 (18.1)	10 (20.8)	20 (18.3)	5 (13.9)	
T3	149 (77.2)	37 (77.1)	86 (78.9)	26 (72.2)	
T4a	4 (2.1)	0 (0.0)	1 (0.9)	2 (8.3)	
Pathological N stage*					<0.001
N0	106 (54.9)	35 (72.9)	64 (58.7)	7 (19.4)	
N1	43 (22.3)	5 (10.4)	24 (22.0)	14 (38.9)	
N2	30 (15.5)	4 (8.3)	16 (14.7)	10 (27.8)	
N3	14 (7.3)	4 (8.3)	5 (4.6)	5 (13.9)	
Pathological TNM stage*					0.001
IB	26 (13.5)	7 (14.6)	17 (15.6)	2 (5.6)	
IIA	80 (41.5)	28 (58.3)	47 (43.1)	5 (13.9)	
IIB	10 (5.2)	2 (4.2)	4 (3.7)	4 (11.1)	
IIIA	36 (18.7)	4 (8.3)	21 (19.3)	11 (30.6)	
IIIB	25 (13.0)	3 (6.3)	15 (13.8)	7 (19.4)	
IIIC	16 (8.3)	4 (8.3)	5 (4.6)	7 (19.4)	
Radiation dose					0.030
<60Gy	52 (33.5)	22 (45.8)	30 (28.0)	–	
≥ 60Gy	103 (66.5)	26 (54.2)	77 (72.0)	–	

* 7th UICC

### Follow up

All patients were assessed at 3-month intervals for the first 2 years after treatment, at 6-month intervals for the next 3 years, and annually thereafter. Computed tomography of the neck, thorax, and upper abdomen using contrast; ultrasonography of the neck and upper abdomen; nuclear bone scanning; conventional blood studies; and biochemistry studies were performed at each follow-up, in addition to gastric endoscopy, positron emission tomography, or cytologic puncture, as needed. Relapses were classified as LRR and distant metastasis (DM). Overall survival (OS) was defined as the interval between recurrence and death from any cause, loss to follow-up, or last follow-up. The disease-free survival (DFS) time was defined as the interval between surgery and first recurrence.

### Statistical analysis

The survival rate was calculated by the Kaplan-Meier method and analyzed using the log-rank test. Multivariate analyses were performed to identify the prognostic factors for post-recurrence OS using the Cox proportional hazards model. All tests were two-sided, and *P* values < 0.05 were considered to indicate statistical significance. All the statistical analyses were performed using the Statistical Package for Social Sciences 23.0 software (SPSS Inc., Chicago, IL).

## Results

### Distribution of resected LNs and failure patterns in locoregional LNs

**Fig. 1:**
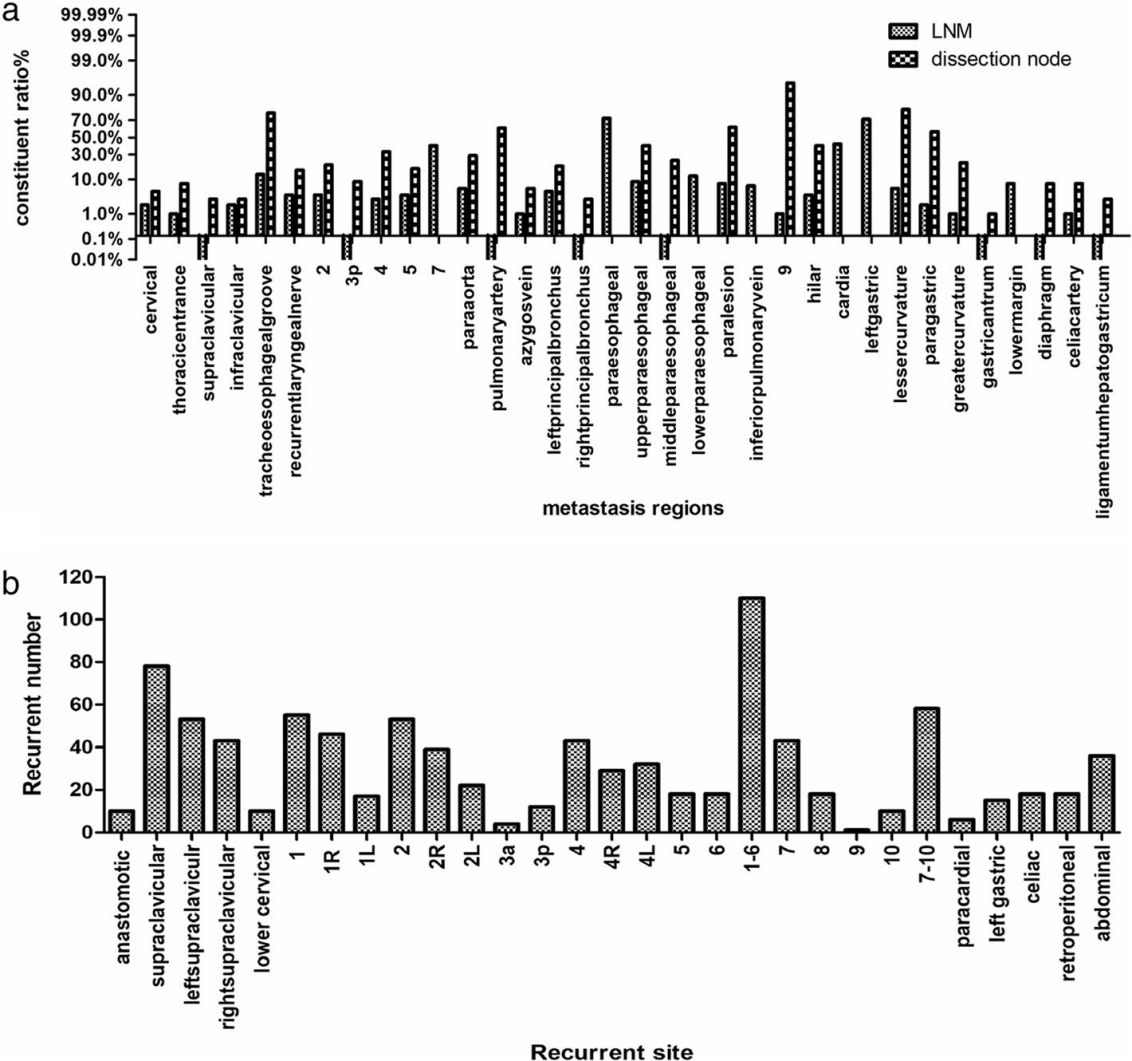
**a** Distribution of lymph node resection (LNM: lymph node metastases); **b** Distribution of recurrent lymph nodes

A total of 148 patients received Sweet esophagectomy and 45 patients underwent the Ivor-Lewis approach. The upper mediastinal LNs were defined as those in the tracheoesophageal groove; with the recurrent laryngeal nerve; at stations 2, 3p, 4, and 5; and with the paraaorta. In total, 201 LNs were resected, including 34 that were confirmed as having metastases (16.9%). Furthermore, 2086 LNs were resected from the paraesophageal and subcarina regions, and only 133 (6.4%) had metastases. The metastasis rate of abdominal LNs was 7.2% (131/1812; Fig. 1a).

Supraclavicular and mediastinal LNs were the most common sites of recurrence (16.6 and 46.6%, respectively). The recurrence rate in the upper abdominal LNs was only 8.8%. Among the 193 patients, 302 sites with LRR were noted, including 36.4% (110/302) of sites in the 1 to 6 mediastinal regional LNs and 25.8% (78/302) of sites in the supraclavicular LNs (Table 2 and Fig. 1b).

**Table 2:** Patterns of recurrence in the locoregional lymph nodes

Site of lymph node recurrence	Number	%
Supraclavicular LN	32	16.6
Mediastinal LN	90	46.6
Upper abdominal LN	17	8.8
Supraclavicular and mediastinal LN	37	19.2
Supraclavicular and upper abdominal LN	3	1.6
Mediastinal and abdominal LN	11	5.7
Supraclavicular, mediastinal, and upper abdominal LN	3	1.6

### Survival rates in different subgroups

The final follow-up was performed on November 24, 2018, with a median follow-up of 96.8 months. The median survival time after recurrence was 13.1 months. The 1-, 3-, and 5-year OS rates after recurrence were 53.5, 22.7, and 17.9%, respectively.

**Fig. 2:**
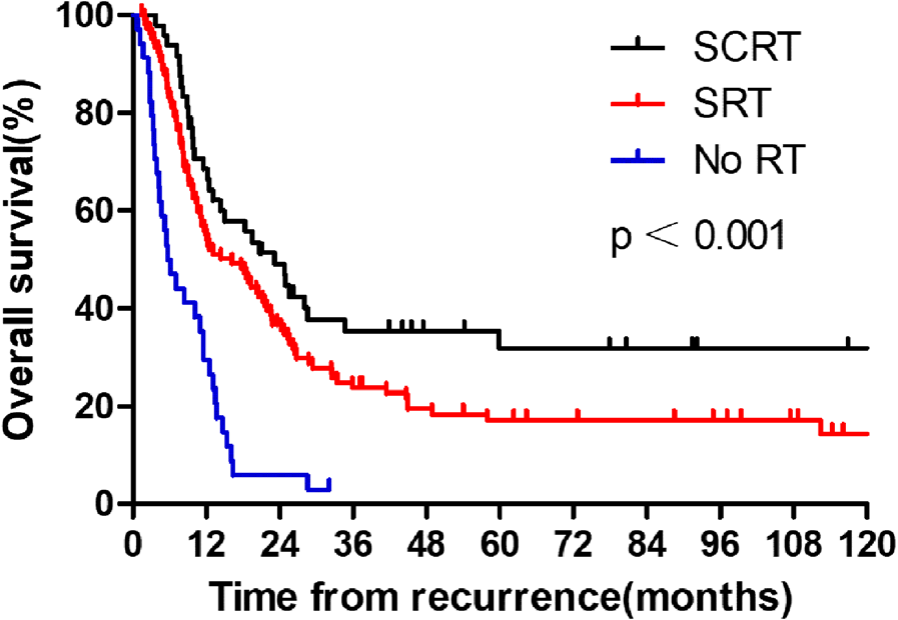
The overall survival rates after recurrence according to different salvage regimens

The median survival time for patients with SCRT, SRT, and No RT were 23.2, 16.2, and 5.7 months, respectively. Moreover, in those with SCRT, SRT, and No RT, the 1-year OS rates were 68.5, 55.0, and 28.6%; 3-year OS rates were 35.4, 23.8, and 2.9%; and 5-year OS rates were 31.8, 17.2, and 0.0%, respectively (*P* < 0.001; Fig. 2). Moreover, there was a significant survival benefit in favor of the SCRT group, relative to the SRT group (*P* = 0.044).

**Fig. 3:**
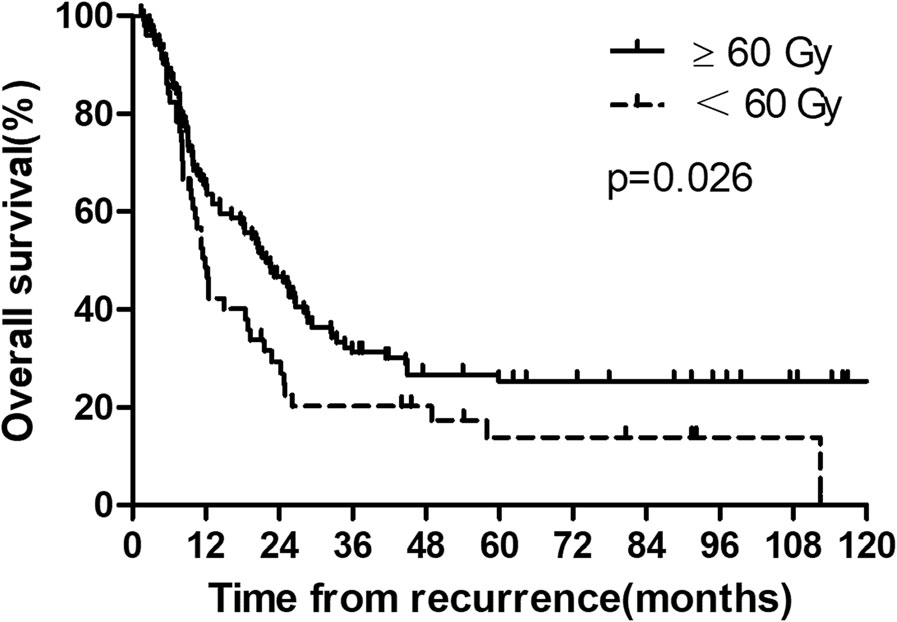
The overall survival rates after recurrence, stratified by the dose of radiation

Two patients were excluded because the radiation dose was unknown. A total of 52 patients received radiation at a dose < 60 Gy, and their median survival time after salvage treatment was 12.0 months; the 1-, 3-, and 5-year OS rates of these patients were 48.5, 20.3, and 13.9%, respectively. Moreover, 103 patients received a dose of ≥60 Gy, and their median survival time was 21.9 months; the 1-, 3-, and 5-year OS rates in these patients were 65.5, 31.2, and 25.3%, respectively. The differences in OS rates between these groups were significant (*P* = 0.026; Fig. 3).

**Fig. 4:**
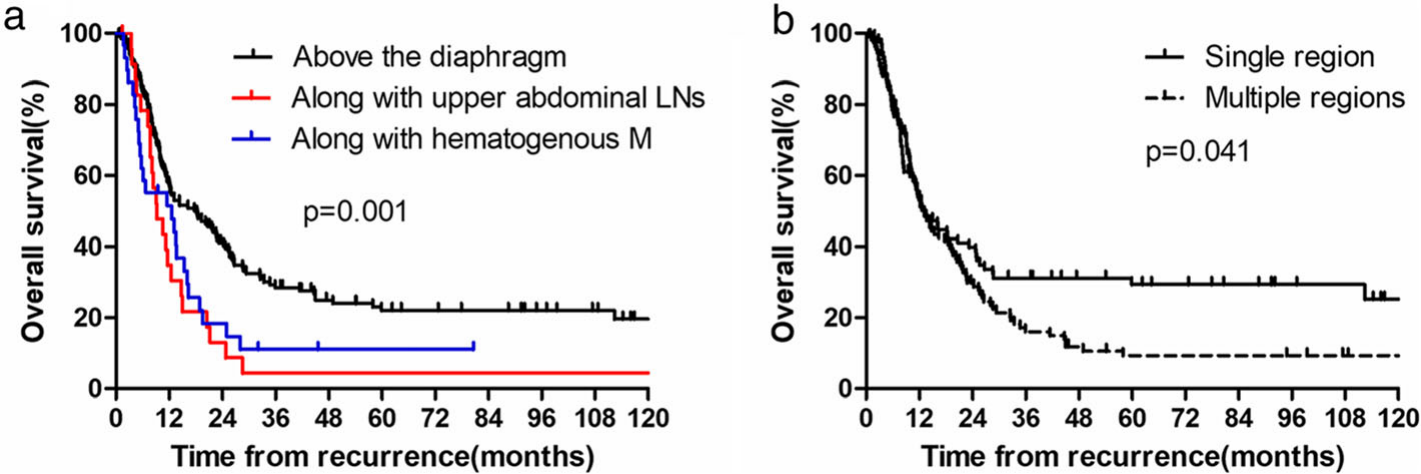
**a** The overall survival rates after recurrence according to the site of recurrence in relation to the diaphragm; **b** The overall survival rates after recurrence based on the number of recurrent LN regions

LN recurrence above the diaphragm was noted in 139 cases, recurrence in the subphrenic LNs was noted in 24 cases, and LN recurrence with hematogenous metastasis was observed in 30 cases; in these 3 scenarios, the 1-year OS rates were 57.5, 34.8, and 49.8%; 3-year OS rates were 28.5, 4.3, and 10.7%; and 5-year OS rates were 22.0, 4.3, and 10.7%, respectively (*P* = 0.001; Fig. 4a). Single region recurrence (SRR) was defined as LN recurrence in only one region, whereas multiple region recurrence (MRR) was defined as relapse in more than 1 LN region. In total, 83 (43.0%) and 110 (57.0%) patients exhibited SRR and MRR, respectively, and the corresponding 1-, 3-, and 5-year OS rates were 54.8, 31.1, and 29.4% for the SRR and 52.6, 15.9, and 9.2% for the MRR cases, respectively (*P* = 0.041; Fig. 4b).

Univariate analysis indicated that the pathological N stage, pathological TNM stage, salvage treatment regimen, recurrence site, and number of recurrent regions were associated with the OS. Furthermore, multivariate analysis showed that the pathological TNM stage and salvage treatment regimen were independent prognostic factors (Table 3).

**Table 3:** Univariate and multivariate analysis for overall survival after recurrence

Factors	Number	Univariate		Multivariate	
		HR(95%CI)	*P*	HR(95%CI)	*P*
Pathological N stage			<0.001		<0.001
pN0	106	1		1	
pN+	87	1.984(1.438–2.737)		0.507(0.360–0.714)	
Pathological TNM stage			<0.001	–	–
I/II	116	1			
III	77	2.353(1.697–3.263)			
Salvage treatment			<0.001		<0.001
SCRT	48	1		1	
SRT	109	1.510(1.002–2.276)	0.049	1.419(0.940–2.142)	0.096
No RT	36	4.175(2.529–6.891)	<0.001	3.246(1.931–5.458)	<0.001
Site of recurrence			0.002	–	–
Above the diaphragm	139	1			
Along with upper abdominal LN recurrence	24	1.967(1.234–3.136)	0.004		
Along with hematogenous metastases	30	1.816(1.177–2.804)	0.007		
Number of LN recurrence regions			0.042	–	–
Single region	83	1			
Multiple regions	110	1.408(1.012–1.958)			

## Discussion

Esophagectomy remains the standard treatment for esophageal cancer. The NCCN guidelines recommend surveillance after radical resection, regardless of the status of primary tumor or regional LN. However, the recurrence rate in these cases remains high, which is a major reason for failure after surgery.

Recent studies have found that supraclavicular and mediastinal LN recurrence are the most common [5, 10–12], although the abdominal LN relapse rate remains at approximately 9.3% [11, 12], consistent with our study. LN dissection around the tracheoesophageal groove and recurrent laryngeal nerve is known to be difficult via Sweet esophagectomy. In the present study, only 23.3% of patients underwent Ivor-Lewis esophagectomy, which reportedly has a higher LN retrieval relative to the Sweet approach, particularly for upper mediastinal LNs [13]. In contrast, the paraesophageal, subcarina, and abdominal LNs are easy to resect via both surgical procedures. Therefore, the bilateral supraclavicular (16.6%) and upper mediastinal (36.4%) regions were the main sites of LN recurrence in our study.

The possible treatments for recurrence include SCRT, surgical resection, SRT, chemotherapy, or best supportive care. The NCCN guidelines recommend SCRT as preferable in patients with LRR who do not receive prior radiotherapy. However, there is a lack of studies with a large sample size on the most effective salvage therapeutic strategies.

The survival rate of salvage treatment is reportedly poor after recurrence. The 1- and 3-year OS rates are approximately 45.9–47.1% and 10.6–17.1%, respectively, whereas the 5-year OS rates are only 4.3–6.4% [14, 15]. In the present study, the 1-, 3-, and 5-year OS rates were 53.5, 22.7, and 17.9% respectively, which are slightly higher than the above-mentioned results. There may be some key reasons for this difference. First, early recurrence detection was the main focus for improving the salvage treatment effect in our study, so that patients could receive prompt treatment. The median time to recurrence was 7.0 months in our study, compared to 13.0–15.0 months in the above-mentioned studies. Second, 83 (43.0%) patients had SRR in our study, and the OS of these cases was markedly higher than that of cases with MRR. Moreover, the present study included approximately 55.0% of patients with original pathological stage IB-IIA, which was more than that (36.3%) in the study of Su et al. [15]. Finally, 81.3% (157/193) patients received SCRT and SRT in our research, whereas only 42.6% (81/190) patients received SRT in the study of Su et al. [15]. Therefore, close follow-up, early recurrence detection, SRR, and timely salvage treatment were vital factors leading to favorable results in the recurrent patients.

The optimal salvage strategies in patients with LRR remain controversial. SCRT was found to confer better survival than SRT in the present study, consistent with that noted in previous studies [16, 17]. Although these studies were retrospective in nature, they support the importance of salvage treatment for LRR, particularly that of SCRT. However, the outcomes of SCRT and SRT after recurrence remain unclear. Nemoto et al. [9] found that SRT combined with chemotherapy had better local control rates but not OS. In contrast, SRT was found to be superior to SCRT in another small sample size study of patients with LRR and DM [18]. In the present study, SCRT had better OS than SRT, and the difference was significant. The possible reason to explain this difference includes the using of three-dimensional conformal radiotherapy in our institution besides above-mentioned early recurrence detection and earlier pathological stage. It has been reported that three-dimensional conformal radiotherapy can increase the local control rate and OS rate in nasopharyngeal carcinoma [19] and lung cancer [20]. Furthermore, chemotherapy with radiotherapy could increase the radiosensitivity [21] and improve OS, relative to radiotherapy alone, in nasopharyngeal carcinoma [22] and esophageal cancer [23]. Thus, SCRT appears to be a superior option for specific patients.

Furthermore, the specific salvage radiation dose differs among various studies. Shioyama et al. [24] assessed 66 patients receiving a dose of ≥50 Gy and 16 patients receiving a dose of < 50 Gy, and observed 2-year and 5-year OS rates of 26.0, 13.0, and 10.0%, 0.0%, respectively (*P* = 0.003); accordingly, the researchers believed that 50 Gy was the best radiation dose for salvage treatment. Zhang and colleagues [25] found that an irradiation dose of ≥60 Gy could improve the OS among patients with recurrent esophageal cancer after surgery (*P* < 0.05). Another retrospective study with a small sample size was not able to determine a significant radiation dose [18]. In the present study, the 5-year OS was 25.3% in the ≥60 Gy group, as compared to 13.9% in the < 60 Gy group (*P* = 0.026). Thus, we believe that a salvage dose of at least 60 Gy might be more reasonable and effective for LRR. However, as our study is retrospective in nature and was performed at a single center, it might suffer from and heterogeneity; hence, a prospective trial may be needed to confirm these findings.

Cases with LN recurrence above the diaphragm exhibited better OS than those with recurrence at the subphrenic LNs and with hematogenous metastasis. Some studies found that the location of regional LN metastases influenced survival, particularly that of celiac LN metastasis [26, 27]. However, research related to abdominal LN recurrence remains insufficient. A retrospective study showed that although the location of LN metastasis did not significantly influence survival, patients with LN metastasis around the abdominal aorta had poor survival [28]. There may be some reasons for this finding. First, LN recurrence under the diaphragm often presents with necrosis and hypoxia, which are associated with poor radiosensitivity. Second, the prescription dose in these cases is insufficient due to limitations of the stomach and intestine. Third, patients with abdominal LN recurrence might be prone to developing hematogenous metastasis. Nevertheless, further research is needed to determine the reason of poor OS for these patients.

In addition, we found that the prognosis of patients with MRR was significantly poorer than that of patients with SRR. Similarly, some studies found that the number of recurrent LNs and number of recurrent LN regions were prognostic factors [2, 7, 28–31]. Researchers considered that patients with multiple recurrent tumors might be considered as having systemic recurrence at the time of diagnosis. This would affect the proportion of patients who receive radiation therapy due to the heavy tumor burden, large target area, and poor status.

**Fig. 5:**
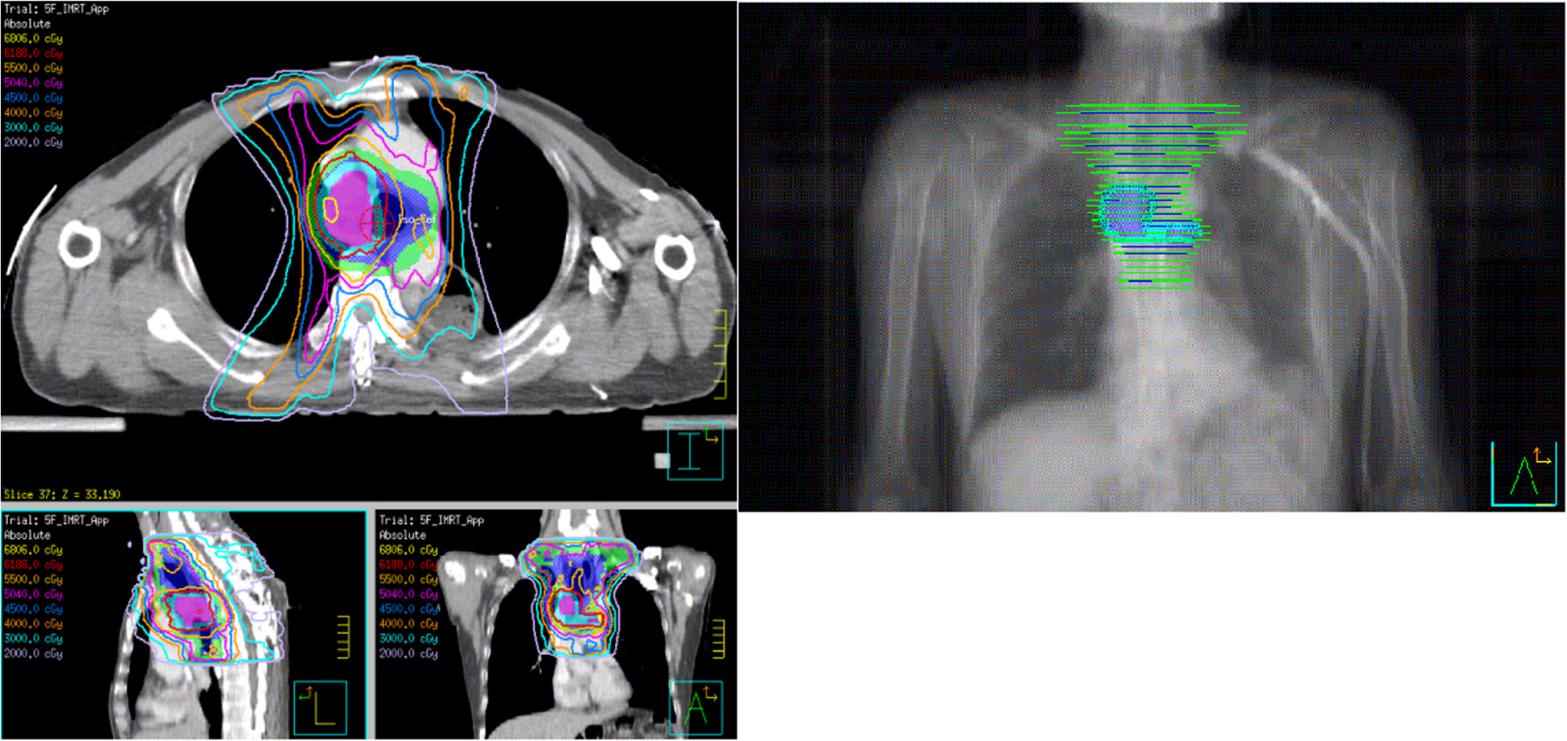
Target contouring and planning design for elective field radiotherapy after recurrence. GTVnd (pink area), CTV (blue area), PGTVnd (sky blue area), PTV (green area). The prescribed dose (SIB-IMRT) for 95% PTV was 50.4 Gy/1.8 Gy/28 f, and for 95% PGTVnd was 61.88 Gy/2.21 Gy/28 f. The concurrent chemotherapy regimen included liposomal paclitaxel (135 mg/m^2^ IV) on day 1 and nedaplatin (50 mg/m^2^ IV) on day 1, which were cycled every 21 days. A total of 2 cycles were delivered

**Fig. 6:**
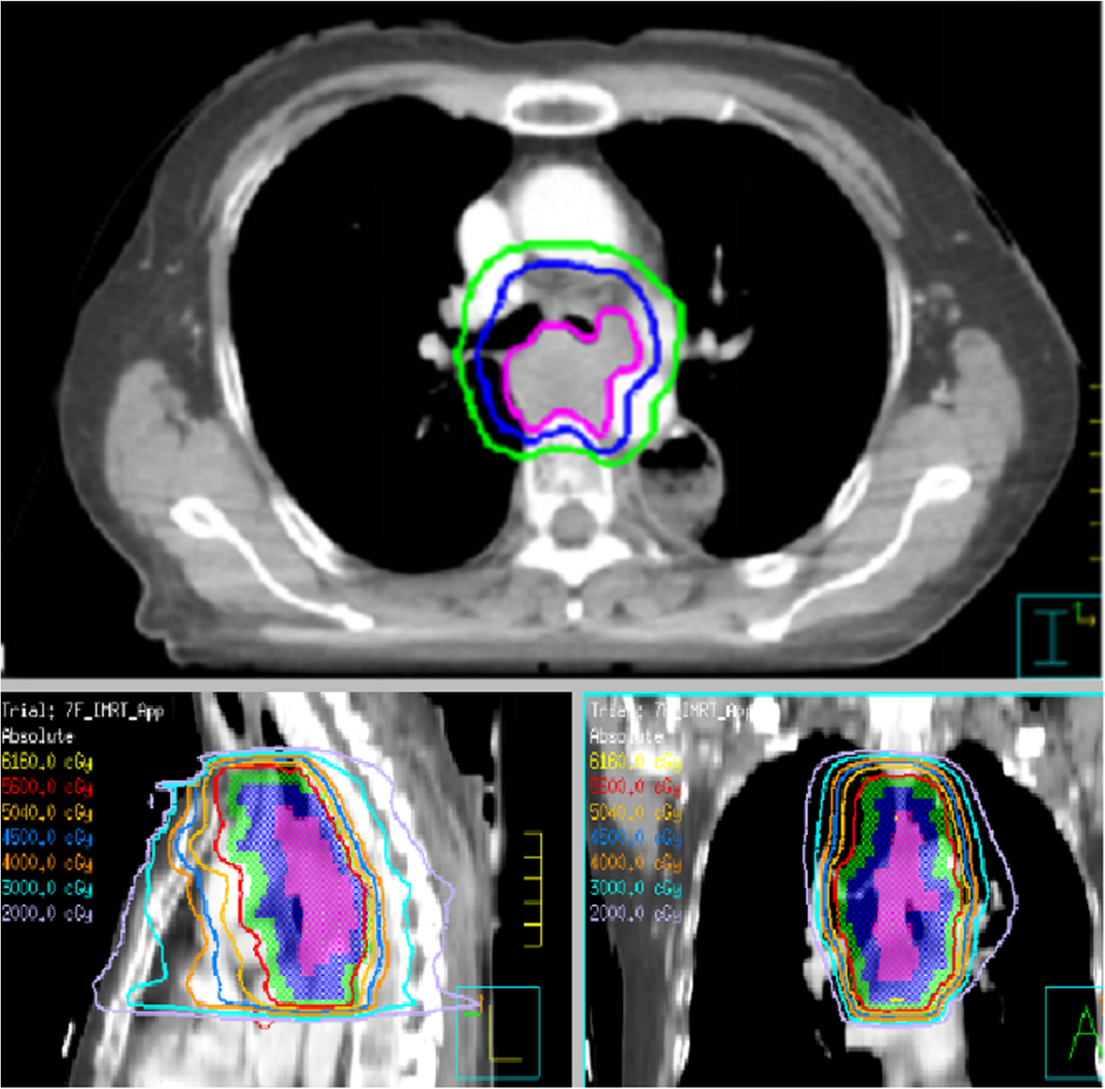
Target contouring and planning design for involved field irradiation. GTVnd (pink area), CTV (blue area), PTV (green area). The prescribed dose (IMRT) for 95% PTV was 56 Gy/2 Gy/28 f

Thus, the clinical findings of the present study suggest that salvage concurrent chemoradiotherapy could not only improve the synergistic effect of radiotherapy, but can also control the potential metastasis of subclinical lesions. The field and dose of radiation should be determined based on the specific recurrence type. Simultaneous Integrated Boost Intensity-Modulated Radiation Therapy (SIB-IMRT) was the optimal irradiation technology to increase the dose of regions at high risk, while simultaneously reducing the dose to organs at risk (OAR; Fig. 5). However, recurrence at anastomotic sites, near anastomotic sites, and near the remnant stomach can be treated with sequential radiotherapy to avoid stenosis of the anastomotic stoma or complications of the remnant stomach. For patients with heavy burden of local recurrence or extensive metastasis at multiple sites who cannot undergo radical treatment, palliative therapy was used to improve the quality of life (Fig. 6).

Multivariate analysis indicated that only the original pathological TNM stage and salvage treatment regimen were independent prognostic factors. Therefore, the salvage treatment regimen is vital in patients with recurrence after radical surgery. We suggest that SCRT or SRT with a dose of ≥60 Gy is the optimal choice for LRR.

The retrospective nature of the present study is a limitation. Moreover, the number of patients across the 3 groups was not balanced. Hence, these findings should be confirmed in a large-scale prospective study.

## Conclusions

Failure was most common in the supraclavicular and upper mediastinal LNs after R0 surgery in TESCC patients. SCRT or SRT with a dose of at least 60 Gy could significantly improve OS in esophageal cancer with locoregional LN recurrence.

## Data Availability

All data generated or analysed during this study are included in this published article.

## Abbreviations

CI: Confidence intervals
CTV: Clinical target volume
DFS: Disease-free survival
DM: Distant metastasis
GTVnd: Metastatic regional nodes
HR: Hazard ratios
IMRT: Intensity-Modulated Radiation Therapy
IV: Intravenous intravenously
LN: Lymph node
LNM: Lymph node metastases
LRR: Locoregional recurrence
MRR: Multiple region recurrence
NCCN: National Comprehensive Cancer Network
OAR: Organs at risk
OS: Overall survival
PGTVnd: Planning metastatic regional nodes
PTV: Planning target volume
SCRT: Salvage chemoradiotherapy
SIB-IMRT: Simultaneous Integrated Boost Intensity-Modulated Radiation Therapy
SRR: Single region recurrence
SRT: Salvage radiotherapy
TESCC: Thoracic esophageal squamous cell carcinoma
UICC: Union for international cancer control
